# Jaburetox: update on a urease-derived peptide

**DOI:** 10.1186/s40409-017-0122-y

**Published:** 2017-06-15

**Authors:** Arlete Beatriz Becker-Ritt, Camila Saretta Portugal, Célia Regina Carlini

**Affiliations:** 1 Graduate Program in Cellular and Molecular Biology Applied to Health, Lutheran University of Brazil (ULBRA), Canoas, RS Brazil; 2 Brain Institute (Instituto do Cérebro-INSCER), Pontifical Catholic University of Rio Grande do Sul (PUCRS), Porto Alegre, RS Brazil

**Keywords:** Peptide, Bacteria, Membranes, Nanoparticles

## Abstract

Urease from *Canavalia ensiformis* seeds was the first enzyme ever to be crystallized, in 1926. These proteins, found in plants, bacteria and fungi, present different biological properties including catalytic hydrolysis of urea, and also enzyme-independent activities, such as induction of exocytosis, pro-inflammatory effects, neurotoxicity, antifungal and insecticidal properties. Urease is toxic to insects and fungi per se but part of this toxicity relies on an internal peptide (~11 kDa), which is released upon digestion of the protein by insect enzymes. A recombinant form of this peptide, called jaburetox (JBTX), was constructed using *jbureII* gene as a template. The peptide exhibits liposome disruption properties, and insecticidal and fungicidal activities. Here we review the known biological properties activities of JBTX, and comment on new ones not yet fully characterized. JBTX was able to cause mortality of *Aedes aegypti* larvae in a feeding assay whereas in a dose as low as of 0.1 μg it provoked death of *Triatoma infestans* bugs. JBTX (10^−5^–10^−6^ M) inhibits the growth of *E. coli*, *P. aeruginosa* and *B. cereus* after 24 h incubation. Multilamellar liposomes interacting with JBTX undergo reorganization of the membrane’s lipids as detected by small angle X-ray scattering (SAXS) studies. Encapsulating JBTX into lipid nanoparticles led to an increase of the peptide’s antifungal activity. Transgenic tobacco and sugarcane plants expressing the insecticidal peptide JBTX, showed increased resistance to attack of the insect pests *Spodoptera frugiperda*, *Diatraea saccharalis* and *Telchin licus licus*. Many questions remain unanswered; however, so far, JBTX has shown to be a versatile peptide that can be used against various insect and fungus species, and in new bacterial control strategies.

## Background

Ureases (urea amidohydrolases; EC 3.5.1.5) are enzymes that catalyze the hydrolysis reaction of urea to ammonia and carbamate, which then decomposes through a spontaneous reaction of carbon dioxide in a second molecule of ammonia. These enzymes have been isolated from a wide variety of organisms including plants, fungi and bacteria [1, 2].

The urease extracted from *Canavalia ensiformis* seeds is one of the landmarks in the study of enzymes. It was the first enzyme to be crystallized, demonstrating that enzymes are proteins [3]. It was also the first one to be identified as a metalloenzyme containing nickel in its active site [4].

The classical urease, called jack bean urease (JBU), is composed of a polypeptide chain of 840 amino acid residues and has a molecular mass of 90 kDa. The minimum active form is a trimer of 270 kDa and it is often found in its native form as a hexamer of 540 kDa [5, 6]. The second isoform of jack bean urease, canatoxin (CNTX), was isolated from the seed and originally characterized as a neurotoxic protein [7]. It features two chains with a molecular mass of 95 kDa held together by non-covalent bonds, and it has about 40% of the enzymatic activity of JBU; each subunit contains one zinc atom and one nickel atom [8]. Importantly, despite the high similarity with JBU, this less abundant isoform has lower ureolytic activity [6].

Ureases and derived peptides show several biological activities including membrane disruption and permeabilization, fungicidal and insecticidal properties [9]. Jaburetox2Ec and jaburetox (JBTX) are the two first versions of a recombinant peptide with 91 amino acids, based on jack bean urease sequence, with a potent effect against insects, yeasts and filamentous fungi [10–12].

Urease and its derived peptide were evaluated for their action on the diuresis of the bug *Rhodnius prolixus* through an in vitro assay with Malpighian tubules [13]. It was observed that although both urease and JBTX inhibited diuresis, they recruited distinct signaling cascades. While urease activates the eicosanoid pathways and depends on the transport of calcium ions, JBTX inhibits diuresis by changes in levels of cGMP and in the transmembrane potential [13].

The insecticidal activity of JBTX occurs at very low doses (0.01 and 0.1% w/w) when compared to other plant derived entomotoxic proteins, regardless of the route of administration, either orally or by injection into the hemocell [12].

Molecular studies of JBTX revealed that the peptide contains a sequence that could adopt a β-hairpin conformation at its C-terminal region, a structure similar to that found in antimicrobial peptides (AMPs) with membrane rupture properties [10]. To assess the importance of the peptide structure in the biological activities of JBTX, Martinelli et al. [14] conducted molecular studies and site-directed mutagenesis, aiming to identify structural motifs related to the toxic activities.

The mutants derived from jaburetox were called: jaburetox N-terminal, corresponding to residues from 1 to 44 (JBTX N-ter); jaburetox C-terminal, residues from 45 to 93 (JBTX C-ter); and jaburetox-Δβ, without the amino acids 61 to 74 (JBTX Δ-β), which corresponds to the β-hairpin region. All mutants were tested in different biological assays. In insect toxicity tests, the data suggest that the β-hairpin region is not important for entomotoxicity and that the N-terminal portion of JBTX is responsible for insecticidal activity. However, the C-terminal region of the peptide, which contains the β-hairpin moiety, is likely to contribute significantly to the ability of JBTX to interact with a lipid bilayer [12, 14].

The ability of these peptides to form ion channels in lipid bilayers was also confirmed by testing with planar lipid bilayers (PLB) [15]. In this study, it was shown that the peptide, as well as the above mentioned mutants can insert themselves into planar lipid bilayers and form cation selective ion channels. The data obtained by Martinelli et al. [14] brought important contributions to the understanding of the JBTX’s mechanism of action, suggesting that it represents a new type of active peptide in membranes, with insecticidal and fungitoxic properties. The antifungal activity of JBTX against yeasts occurs at higher doses (9 to18 μM) than those observed for JBU (0.27 μM), suggesting that other regions of the protein could probably be involved in this activity [11].

The overall conformation of the peptide JBTX was elucidated using techniques such as light scattering, circular dichroism and nuclear magnetic resonance [16]. The authors demonstrated the intrinsically disordered nature of the peptide, which exists in a “pre molten globule” state, and its tendency to form an α-helix motif near the N-terminus and two turn-like structures (located in its central/C-terminal polypeptide portions). Thus, although JBTX has low propensity to present secondary structure, and despite being an intrinsically disordered protein, the peptide has some degree of folding [16].

## What’s New?

### Can the peptide control disease vectors and insect pest in agriculture?

The mosquito *Aedes aegypti* (Diptera: Culicidae) is the vector responsible for transmitting diseases to humans, such as urban yellow fever, dengue and more recently, Chikungunya and Zika viruses [17]. Presently the main form of control of these diseases still is by fighting its vector, which requiers complex and coordinated actions of various sectors of the society as well as changes in population habits [18].

In Brazil, endemic disease fighting agents and national and municipal health agents are working together with the population and are responsible for promoting the chemical mechanical control of the disease vector. The actions are focused on detecting and destroying natural or artificial reservoirs of water that can serve as a deposit for the mosquito’s eggs. Educational activities are another strategy postulated by the Ministry of Health including community agents visiting residences, in order to ensure the sustainability of the elimination of breeding sites, in an attempt to break the transmission chain of the disease [17].

**Fig. 1:**
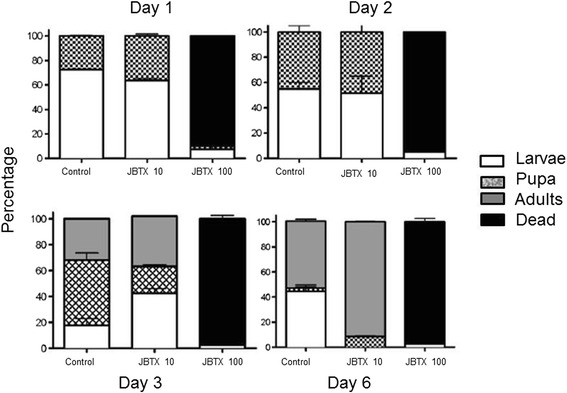
Effects of jaburetox-V5-loaded lyophilized *E. coli* on *A. aegypti*. Mosquito larvae, 25 per experimental condition, were fed fish diet soaked with a suspension of lyophilized *E. coli* cells containing 0 (control), 10 or 100 μg of JBTX, as quantified by ELISA. Controls with non-transformed *E. coli* were run in parallel. The larvae were kept at 28 ^o^C with a photoperid of 8:16 (light:dark) and developmental stage and mortality were recorded daily up to day 6. The results are average of duplicates, and expressed as a percentage relative to the initial number of insects. Copyright by K. Kappaun [19]. Reprinted with permission

In order to test a new mosquito control strategy, the insecticidal activity of lyophilized *Escherichia coli* cells overexpressing the peptide JBTX against *A. aegypti* larvae was evaluated [19]. Peptide quantification performed by ELISA showed that *E. coli* cells produced about 27 μg JBTX per mg of dry matter. For the test, mosquito larvae were feed with fish food containing the recombinant *E. coli* cells to 10 or 100 μg of JBTX. As control, *E. coli* cells without JBTX were used in the same proportion. The survival rate and stage of progression of the biological cycle from pupa to adult were accompanied. A suspension of *E. coli* containing 100 μg of JBTX promoted 90% mortality of *A. aegypti* larvae on the first day and 97.5% in 6 days Fig. 1. The results emphasized the entomotoxic potential of JBTX to control *A. aegypti* by interfering in the mosquito biological cycle and producing mortality of larvae as well as adult insects [19].

*Triatoma infestans* is the main vector of Chagas disease in South American countries and the control of the disease strongly depends on vector eradication [20]. Despite extensive insecticide application, the disease is still endemic in South America [21]. Using *T. infestans* as a model, Galvani et al. [22] demonstrated that injection of 0.1 μg of JBTX/mg of body weight into adults caused death of all insects in less than 24 h. After 3 h of injection, insects show neurotoxic symptoms such as abnormal behavior of antennae and uncoordinated leg movements, which precede death. JBTX was found to bind to neuronal cells and to interfere with at least two enzymes of the insect brain:

- JBTX strongly inhibited the activity of nitric oxide synthase, thereby reducing the levels of the nitric oxide neurotransmitter;
- JBTX was found to physically interact with and to increase the activity of UDP-N-acetylglucosamine pyrophosphorylase (UDP-GlcNAcP), an enzyme involved in glycosylation pathways and in the synthesis of chitin [22].

In an in vitro study, JBTX also activated UDP-GlcNAcP from the cotton stainer bug *Dysdercus peruvianus*, an insect that is susceptible to the insecticidal activity of both, urease and JBTX [22, 23].

*Rhodnius prolixus*, another triatomine vector of Chagas disease in South America, has been extensively used as an insect model in the studies to elucidate the mechanism of action of urease and derived peptides. Besides interfering with diuresis, crop physiology and causing effects related to the central nervous system (CNS), JBTX was shown to disrupt the immune response of *R. prolixus*, affecting the ability of the insect to effectively counteract bacterial infection [24].

According to the United Nations data, about one billion people are still hungry worldwide [25]. In addition, according to the World Hunger [26], almost all the hungry people are living in developing countries. Globally, every year about 35% of all crop production is lost to pre-harvest biotic stresses and an additional 6 to 20% of losses are due to post-harvest events [27]. With an average annual loss of 25 million tons, corresponding to 7.7% of the Brazilian agricultural production, the financial damage can reach $ 16 billion per year [28].

If we take only into account the sugarcane crop, Brazil is the largest producer in the world. Data from the National Supply Company (Conab) show that in the 2016/2017 harvest, the country harvested over 657 million tons of sugarcane with a production of 38 million tons of sugar [29]. The losses that the giant borer, *Telchin licus licus*, can potentially cause include reduction of 12.1% in sugarcane production, 4% loss in sugar production and 3% reduction in ethanol production. Moreover, annual expenditures on insect control methods allow estimating that the losses caused by this insect can reach R$ 4.88 billion per year [29].

To access the effect of JBTX on the cotton stainer bug *Dysdercus peruvianus* an insect model that relies on cathepsins as its main digestive enzyme was employed. Third instars insects were fed artificial cottonseeds containing lyophilized 0.01% (w/w) JBTX. After 10 days, the mortality was two times higher for bugs that fed on freeze-dried purified JBTX than that observed for those that ingested the jackbean urease isoform canatoxin, at the same dose [10, 23].

Insects relying on trypsin-like alkaline serine-proteinases as main digestive enzymes, such as the fallworm *Spodoptera frugiperda*, were shown to be resistant to canatoxin’s insecticidal effect. This fact was attributed to the breakdown of canatoxin by the proteolytic enzymes produced by these insects as well as to the lack of production of the entomotoxic peptide. On the other hand, third instar *S. frugiperda* reared on *Phaseolus vulgaris* foliar discs containing air-dried JBTX were susceptible to the entomotoxic activity of JBTX. These experiments demonstrated that lepidopterans and other insects relying on trypsin-based digestion, although not being able to hydrolyze urease to release its internal peptide, could be targets of the preformed toxic peptide [10, 23].

Another example is that of the polyphagous pest *Helicoverpa armigera* (corn earworm). It was first identified in Brazil during the 2012–2013 crop season, causing serious damage to the production of cotton, soybean, corn, green beans, tomatoes, citrus and pastures. Asia, Europe, Africa, and Australia reports U$ 2 billion damages caused by *H. armigera* annually, whereas Brazil suffered a damage of approximately U$ 0.8 billion when it first emerged [30]. Feeding on a few micrograms of JBTX, or its truncated version with deletion of a β-hairpin, caused mortality and a significant reduction in dietary intake in *Helicoverpa armigera* caterpillars [14]. When newborn caterpillars were fed on disks of corn leaves containing the peptides, 69% mortality and a 70% reduction in consumption were observed (Didoné et al., unpublished data).

## Can bacteria be controlled using the peptide?

Bacteria are responsible for causing heavy agricultural losses and for the vast majority of hospital infections [31]. The USA expends per year about 30 billion dollars dealing with hospital infections, and this amount is expected to increase as more bacteria become drug resistant [31].

**Fig. 2:**
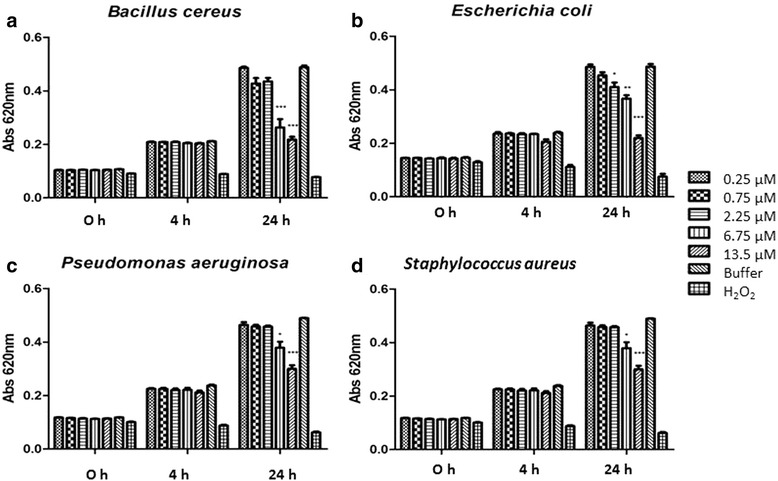
Effects of jaburetox on bacteria. Bacterial multiplication was evaluated by absorbance at 620 nm at time zero, and 4 and 24 h after incubation in the presence of different concentrations of JBTX. **a** *Bacillus cereus*; **b** *Escherichia coli*; **c** *Pseudomonas aeruginosa*; **d** *Staphylococcus aureus*. Growth in the presence of buffer (Tris HCl 10 mM pH 7.0) or H_2_O_2_ was considered as negative and positive control, respectively. Each graph represents three independent experiments in triplicates for each condition. Results are mean ± SD. Asterisks (*) indicate statistical differences (*p* values ≤ 0.05, Tukey test). Copyright by I. A. Terra [33]. Reprinted with permission

Following the method described by Pompilio et al. [32], JBTX – at a wavelength of 620 nm and turbidimetrically monitored – revealed a bacteriostatic effect against *Bacillus cereus*, *Escherichia coli*, *Pseudomonas aeruginosa* and *Staphylococcus*. Bacteria were incubated with different concentrations of JBTX (from 0.25 up to 13.5 μM) added to the growth medium. Their multiplication rate was compared to that in the presence of buffer (Tris HCl 10 mM, pH 7.0) as a negative control and H_2_O_2_ as a positive control. Figure 2 shows a dose-dependent inhibitory effect of JBTX on the four bacterial strains. Growth inhibition of 50% was seen for *E. coli*, *P. aeruginosa* and *B. cereus* with 13.5 μM of JBTX after 24 h of incubation [33]. This inhibitory effect was reversed upon transferring the bacteria to a JBTX-free medium (not shown).

In the context of plant-derived antibacterial peptides, JBTX is as effective as other molecules described in the literature, as summarized in Table 1. Thus fabatins show bacterial activity against gram-negative bacteria in the range of 4–20 μM [34, 35]. Cp thionine-2 is active at 12–25 μM concentration [36]. Hispidalin, from the seeds of *Benincasa hispida*, at 7-μM concentration demonstrated a broad inhibitory effect against bacteria and caused significant inhibition of the filamentous fungi [37].

**Table 1:** Antimicrobial activity of some peptide classes

AMP class	AMP name	Plant species	Antimicrobial activity	Reference
Defensin	Cp-thionin-2	*Vigna unguiculata*	*Staphylococcus aureus* (128 μg · mL^−1^) *Escherichia coli* (64 μg · mL^−1^)	[36]
Defensin	Fabatin-1	*Vicia faba*	*E. coli* (100 μg · mL^−1^) *Pseudomonas aeruginosa* (30 μg · mL^−1^)	[35]
Defensin	Fabatin-2	*V. faba*	*E. coli* (100 μg · mL^−1^) *P. aeruginosa* (30 μg · mL^−1^)	[35]
Defensin	*StPTH1*	*Solanum tuberosum* cv Jaerla	*Clavibacter michiganensis* (7 μM) *Ralstonia solanacearum* (25 μM) *R. solanacearum* (*rfa* ^-^) (25 μM) EC_50_ – Effective concentration for 50% inhibition	[44]
Defensin	Cn-AMP1	*Cocos nucifera*	*Bacillus subtilis* (76 μg · mL^−1^) *S. aureus* (80 μg · mL^−1^) *E. coli* (82 μg · mL^−1^) *P. aeruginosa* (79 μg · mL^−1^)	[45]
Defensin	Cn-AMP2	*C. nucifera*	*B. subtilis* (150 μg · mL^−1^) *S. aureus* (170 μg · mL^−1^) *E. coli* (170 μg/mL) *P. aeruginosa* (169 μg · mL^−1^)	[45]
Defensin	Cn-AMP3	*C. nucifera*	*B. subtilis* (257 μg · mL^−1^) *S. aureus* (274 μg/mL); *E. coli* (302 μg · mL^−1^) *P. aeruginosa* (259 μg · mL^−1^)	[45]
Cyclotide	Kalata B1	*Oldenlandia affinis* (Roem. & Schuld.) DC.	*S. aureus* (0.75 μg · mL^−1^) and *Klebsiella oxytoca* (158.37 μg · mL^−1^)	[46]
Cyclotide	Circulin A	*C*ollinsia *parvifolia* Schum.	*S. aureus* (0.59 μg · mL^−1^) and *Proteus vulgaris* (172.04 μg · mL^−1^)	[46]
Cyclotide	Circulin B	*C. parvifolia* Schum.	*S. aureus* (44.32 μg · mL^−1^), *E. coli* (1.35 μg · mL^−1^), *P. aeruginosa* (83.7 μg · mL^−1^), *P. vulgaris* (22.3 μg · mL^−1^) and *K. oxytoca* (26.92 μg · mL^−1^)	[46]
Cyclotide	Cycloviolacin O2	*Viola odorata*	*Salmonella enterica* (8.75 μg · mL^−1^) *E. coli* (2.2 μg · mL^−1^) *S. aureus* (>50 μg · mL^−1^)	[47]
α/β-Thionin	Alpha-1-purothionin	*Triticum aestivum*	*Pseudomonas solanacearum* (5 μg · mL^−1^) *Xanthomonas phaseoli* (27 μg · mL^−1^) *Xanthomonas campestres* (56 μg · mL^−1^) *Erwinia amylovora* (540 μg · mL^−1^) *Corynebacterium flaccumfaciens* (110 μg · mL^−1^) *Clavibacter michiganense* (450 μg · mL^−1^) *Corynebacterium poinsettiae* (56 μg · mL^−1^) *Corynebacterium sepedonicun* (1 μg · mL^−1^)	[48]
α/β-Thionin	PR-13 thionins	*Nicotiana attenuate*	*Pseudomonas syringae* pv. *Tomato* (0.25 μg · mL^−1^)	[49]
Snakin	Snakin-1	*S. tuberosum*	*Listeria monocytogenes* (10 μg · mL^−1^)	[50]
Snakin	StSN1	*S. tuberosum* cv Jaerla	*C. michiganensis* (4 μM) *R. solanacearum* (*rfa* ^-^) (15 μM) EC_50_ – Effective concentration for 50% inhibition	[44, 51]
Snakin	StSN2	*S. tuberosum* cv Jaerla	*Clavibacter michiganensis* (1 μM) *R. solanacearum* (*rfa* ^*-*^) (30 μM) *Rhizobium meliloti* (8 μM) EC_50_ – Effective concentration for 50% inhibition	[44, 51]
LTP	LTP-s1 LTP-s2	*Spinacia oleracea*	*C. michiganensis* subsp. *Sepedonicus* (100 μg · mL^−1^)	[51]

The yet preliminary evaluation of the antibacterial activity of JBTX demonstrated its inhibitory activity against bacteria of medical and agricultural importance at doses ranging from 2.25 μM (for *E. coli*) to 6.75 μM (*B. cereus*, *P. aeruginosa*, *S. aureus*). The antibacterial activity of JBTX reinforces previous findings of antimicrobial activity of this plant-derived peptide against fungi and yeast of biomedical importance [11]. Therefore, jaburetox is a promising lead molecule for development of new antibiotics and antifungal drugs (Fig. 2) [33].

## Is there any effect on membranes?

JBTX’s ability to interact with lipid membranes has been previously described. Barros et al. [38] observed that JBTX was able to permeabilize acidic liposomes to release entrapped carboxy-fluorescein. Piovesan et al. [15] reported that JBTX, as well as some of its truncated mutants, was capable of inserting itself into neutral planar lipid bilayers forming cation-selective ion channels. More recently, Micheletto et al. [39] studied by small angle X-ray scattering (SAXS) the interaction among JBTX and multilamellar liposomes with a lipid composition typical of human platelets membrane. The interaction among JBTX and the liposomes led to alterations of the Bragg peak, indicating a significant reduction of the lamellar repeat distance and in the number of lamellar repeats. The data suggested that, besides not causing lysis of the vesicles, JBTX promoted a reduction in the size of the liposomes probably due to reorganization of the membrane lipids in the presence of the peptide. A reduction of the Caillé parameter indicated that the liposome membrane became more rigid, which altered the peptide insertion into the lipid membrane.

Interestingly, jack bean urease (JBU), from which JBTX is derived, showed similar behavior towards platelet-like multilamellar liposomes. Since the JBU internal sequence corresponding to JBTX is well exposed at the protein surface, we hypothesized that the JBTX-equivalent region of JBU drives the interaction of the protein with membranes [15]. The data revealed that indeed JBTX can insert itself into a lipid bilayer, eventually traversing the membrane, thereby disrupting the multilamellar structure of the liposomes [39]. There is no data so far to explain why or how JBTX can be selective towards pathogens or pests, while sparing host cells. In fact, JBTX has proved not to be cytotoxic against a panel of mammal tumoral cells at concentrations in the micromolar range (Portugal et al., unpublished data).

## Are JBTX-expressing transgenic plants more resistant to insect pests?

With the exponential growth of the world population and the accompanying need for increasing food production, the advance of agriculture implies the creation of new insect control technologies that protect plants both during their development and at post-harvest (storage). The promise of transgenic crops expressing insecticidal polypeptides that dates back from the 1970s is now becoming true [40]. Corn plants producing Cry proteins derived from *Bacillus thuringiensis* (Bt) soil bacterium are produced since the early 1990s [41]. It is estimated that around 60 million hectares of Bt maize are grown globally and of these, 13 million hectares are cultivated in Brazil alone [42]. However, because insect resistance to the present Bt-expressing crops rapidly evolves, there is an urgent need to find new insecticidal polypeptides for the next generation of pest resistant transgenic plants to be used alone or in “stacking” strategies.

Preliminary biosafety studies showed that high doses of JBTX are innocuous to mice and rats, administered either orally or via injection. This finding encouraged studies on how to develop insect-resistant transgenic plants by heterologous expression of JBTX. A first attempt was conducted by Mulinari in 2008 [43], in which the peptide was inserted into the genome of tobacco SR1 plants by using a binary vector pCAMBIA2300-AMV-35Sd-*jaburetox*2-Tnos through *Agrobacterium* transformation. Young leaves of different transgenic tobacco plants expressing different levels of JBTX (as measured by ELISA) were then offered to *Spodoptera frugiperda* larvae. While leaves of some plants induced 50% lethality of caterpillars (20 larvae per condition – plants with different amount of JBTX) after 30 days, other plants killed 100% of the larvae after 15 days [43].

In preliminary studies, transgenic sugarcane plants (cultivar SP803280) expressing the JBTX peptide under the 35S promoter were obtained by callus bombardment. About 22 PCR-positive clones were generated and after regeneration, the transgenic sugarcane plants expressing JBTX (0.35 to 0.65 μg per mg of total protein, quantified by ELISA) are now being screened for insect resistance. When challenged with *Diatraea saccharalis*, the stem borer, several of these plants proved to be more resistant to the caterpillar attack and in some cases caterpillar mortality reached 100% (Becker-Ritt et al., unpublished data). The JBTX-expressing plants were also tested for resistance against the giant borer, *Telchin licus licus*, a relevant pest of sugarcane crops in some parts of Brazil. The young caterpillar feeds initially on the leaves of sugarcane and then penetrates through the soft parts of the stem (sheath). In some cases, when transgenic sugarcane plants expressing JBTX were exposed to giant borer larvae, 100% lethality of caterpillars was observed (Becker-Ritt et al, unpublished).

## Thinking about the future

While diseases transmitted by *A. aegypti* continue to cause many deaths and insect pests damage livestock and agricultural production, there will be room for research on new and promising insecticidal agents such as JBTX. Not to mention the potent antifungal and bactericidal properties of this urease-derived polypeptide. However, many questions remain and should be clarified before any potential use of JBTX can be devised.

The mechanism of action of JBTX should be better understood. Is the ability of the peptide to interact with lipids what drives its interaction with cells or is there a “receptor” for the peptide in insect membranes? What happens with the intrinsically disordered regions of the peptide when it interacts with biological membranes? Does it acquire a more ordered biologically active structure or is its disordered nature required for its effects? How exactly does JBTX exert its neurotoxic effects or its immunomodulatory action? How specific is JBTX towards insects? Would the biosafety profile of JBTX allow the continuation of studies with insect resistant transgenic plants? Can nanoparticle technologies applied to JBTX, which aim at a controlled and efficient delivery of the entomotoxic peptide to its targets, provide novel solutions to overcome insect resistance, protect the environment and improve crop production? These are only a few questions awaiting to be answered. Meanwhile, although putting all our efforts to unravel the mysteries of JBTX, we still feel mesmerized on the wonders of this beautiful and versatile molecule.

## Conclusions

Our goal with this article was to briefly review the biological activities performed by a recombinant peptide obtained from the *Canavalia ensiformis* urease sequence. The idea was to demonstrate that this recombinant peptide is capable of exerting inhibitory activity on fungi, yeasts, bacteria and insects, and similarly to other plant ureases, JBTX is also capable of acting on plant defense. In addition, the peptide is effective in inhibiting bacteria of medical and agronomic interest and is capable of causing lethality in insect pests of sugarcane, tobacco and corn. In spite of these biological activities, the peptide is innocuous to mammals and non-target organisms and can be used both in the development of transgenic plants resistant to diseases and pests and in the formulation of bioinsecticides.

## Abbreviations

AMPs: Antimicrobial peptides
Bt: *Bacillus thuringiensis*
CNS: Central nervous system
CNTX: Canatoxin
H_2_O_2_: Hydrogen peroxide
Jaburetox-Δβ: Jaburetox without the amino acids 61 to 74
JBTX: Jaburetox
JBTX C-ter: Jaburetox C-terminal
JBTX N-ter: Jaburetox N-terminal
JBU: Jack bean urease
PLB: Planar lipid bilayers
SAXS: Small angle X-ray scattering
UDP-GlcNAcP: UDP-N-acetylglucosamine pyrophosphorylase
